# Detecting Communities Based on Network Topology

**DOI:** 10.1038/srep05739

**Published:** 2014-07-18

**Authors:** Wei Liu, Matteo Pellegrini, Xiaofan Wang

**Affiliations:** 1 Department of Automation, Shanghai Jiao Tong University, Shanghai, 200240, China; 2 Department of Molecular, Cell and Developmental Biology, University of California, Los Angeles, CA, 90055

## Abstract

Network methods have had profound influence in many domains and disciplines in the past decade. Community structure is a very important property of complex networks, but the accurate definition of a community remains an open problem. Here we defined community based on three properties, and then propose a simple and novel framework to detect communities based on network topology. We analyzed 16 different types of networks, and compared our partitions with Infomap, LPA, Fastgreedy and Walktrap, which are popular algorithms for community detection. Most of the partitions generated using our approach compare favorably to those generated by these other algorithms. Furthermore, we define overlapping nodes that combine community structure with shortest paths. We also analyzed the *E. Coli.* transcriptional regulatory network in detail, and identified modules with strong functional coherence.

Communities are groups that are densely connected among their members, and sparsely connected with the rest of the network. Community structure can reveal abundant hidden information about complex networks that is not easy to detect by simple observation. There are many large-scale complex networks (systems) in the real world whose structure is not fully understood. A great deal of research has been carried out to uncover the structures of these real world networks, to improve the ability to manage, maintain, renovate and control them. With the help of varied approaches, it is possible to shed light on the general structure of these networks, and further understand their function.

Network science methods have been used in various settings^1^,^2^, including social^3^,^4^, information^5^, transportation^6^, energy^7^, ecological^8^, disease^9^, and biological networks^10^,^11^,^12^,^13^. In most of these cases we can find clear community structures, which are usually associated with specific functions. However, to date, most detection methods have limitations, and there is still a lot of room to develop more general approaches.

At present, most methods focus on the detection of node community. One popular approach is based on the optimization of the modularity Q^14^,^15^,^52^,^56^ of a sub-network. Some methods^13^,^14^,^29^,^34^,^38^,^39^,^40^ force every node to be assigned to a single community. This assumption doesn't always reflect real world networks, where several overlapping communities can co-exist. For example, in social networks, a person may have family relationship circles, job circles, friend circles, social circles, hobby circles and so on. Algorithms that can discover overlapping communities^16^,^17^,^18^,^19^,^20^,^21^,^22^,^23^ have been developed, and recently, methods to detect link communities^20^,^24^,^25^ have been presented. The concept of a link community is useful for discovering overlapping communities, as edges are more likely to have unique identities than nodes, which instead tend to have multiple identities. In addition, statistical^54^, information-theoretic^35^,^48^,^53^ and synchronization and dynamical clustering approaches^49^,^50^,^58^,^59^,^60^ have also been developed to detect communities.

No matter which method is used to detect community structure, they should present a quantitative definition of community first, but no definition is universally accepted so far. Here we defined community based on three properties, and then propose a simple and novel method to detect communities based on network topology. The main idea is similar to “fishing”. We first use the adjacency lists of nodes (network topology) as a “fishpond”, then detect some strong sense communities from “fishpond” as “baits”, and then use these “baits” to catch the “fish” (weak sense communities). Our method is simple, stable, and easy to understand. It's a parameter free approach, and it can detect overlapping communities, isolated communities and determine the number of communities in an unsupervised manner. Moreover, our method can detect not only cohesive and large communities, but also sparse and small ones. We analyzed 16 different types of networks with our method and the results show that our approach compares favorably to other methods.

## Results

We have tested the performance of our method with both synthetic networks and real-world networks. The size of the networks spans tens to tens of thousands of nodes. We analyzed 16 different types of networks (as shown in table 1), and compared our partitions with several popular algorithms. The results are in tables 2 and 3. Below we analyze one synthetic network and four real world networks in detail.

### Synthetic Network

**Figure 1:**
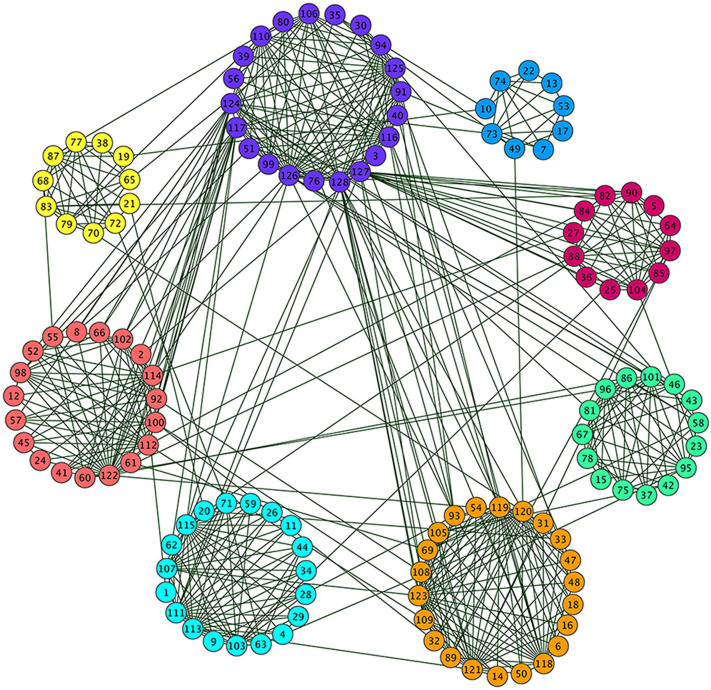
A network of LFR-benchmark with 128 nodes. This network is constructed from 8 pre-assigned communities, and 10 overlapping nodes are included.

The synthetic network we analyzed is the LFR benchmark network^42^. It contains 128 nodes and 597 edges, and includes 8 communities and 10 overlapping nodes. The average degree is 9.328, the maximum degree is 30, the minimum size of a community is 10, the maximum size of a community is 30, the mixing parameter is 0.1, and the number of memberships of overlapping nodes is 2. As shown in Fig. 1, we detected 8 communities in this network using our algorithm, and they are identical with those real communities, except for the 10 overlapping nodes. The reason is these 10 overlapping nodes do not satisfy the overlapping node definition of our algorithm, which combines the structure property with shortest path. We provide examples of other benchmark networks in table 2.

### Real-World Networks

**Figure 2:**
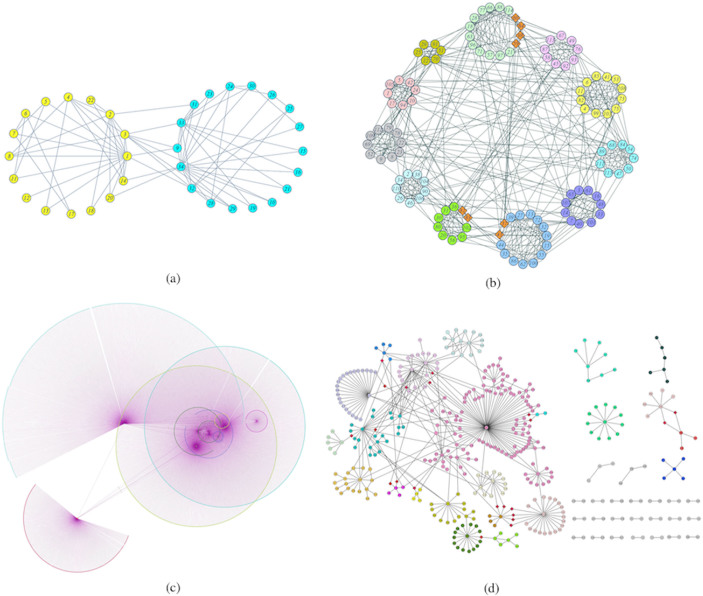
The networks of real-world. (a) Zachary karate club network: the two communities we detected are identical with the two real communities. (b) US college football network: the 11 communities we detected are identical with the 11 real conferences except for the 8 independent teams, which are assigned to 3 different conferences respectively. (c) Facebook network: we discover 7 communities and 9 overlapping nodes (orange diamond shaped nodes) using our algorithm. (d) *E. Coli* transcriptional regulatory network: we discovered 46 modules, 5 isolated nodes and 19 overlapping nodes (red diamond shaped nodes) with our algorithm.

The Zachary karate club network is a famous empirical network. A conflict between club president John (node 34) and the instructor Mr. Hi (node 1) lead to 34 members of the university sports club to split into two groups^30^. As Fig. 2 (a) shows, the two communities discovered by our algorithm are identical with the groups described by Newman^30^. We note that some nodes should be defined as “overlapping nodes” based on the general definition. For example, node 10 and node 3 have equal numbers of connections with two communities, but they have different shortest path lengths with nodes 1 and 34, the hub nodes. As a result, we don't define them as overlapping nodes based on our definition.

The American college football network is another widely used empirical network compiled by Newman^14^ in 2004. There are 115 Division I-A teams that play 613 games during the regular fall season of 2000, and these teams are grouped into 11 different conferences, except for 8 independent teams. We found 11 strong sense communities using our algorithm, as shown in Fig. 2 (b). These are identical to the 11 conferences of Division I-A teams, except for the 8 independent teams, which are assigned to “The Southeast Conference”, “The Big East Conference” and “The Mid-American Conference” respectively.

The Facebook network^34^ is a directed user–user friendships network compiled by Julian and Jure in 2012. There are 2888 users and 2981 friendships in this network. An edge indicates that the user represented by the left node is a friend of the user represented by the right node. We identified 7 strong sense communities and 9 overlapping nodes, as shown in Fig. 2 (c). The structure of this network is very clear based on visual inspection. All the friendships are established around ten users, so that we should have 10 communities. Our partitions merge community pairs involving nodes 603 and 288, 710 and 714, and 2687 and 2699. We found that all these pairs are directly connected to each other and all the overlapping nodes are of the first type.

### Biological Network

The E. Coli transcriptional regulatory network^33^ was compiled by Shen-Orr et.al. in 2002. There are 423 operons and 519 regulatory links as well as 5 self-regulation events. This is a directed network, and each edge is directed from an operon that encodes a transcription factor to an operon that it directly regulates (an operon is one or more genes transcribed on the same mRNA). Here we use an undirected version of the network, and analyze the network using the updated RegulonDB^47^ 8.3.

As shown in Fig. 2 (d), the E. Coli transcriptional regulatory network is composed of 29 disconnected sub-networks and 5 isolated nodes. We detected all the disconnected parts and isolated nodes correctly using our algorithm. The largest sub-network was divided into 18 communities and 19 overlapping nodes. We analyze the 23 modules that have more than 3 members using the DAVID functional annotation tool^45^,^46^. All of the communities are functionally coherent (i.e. the genes appear to participate in a common biological process). For example, the first module contains 7 operons, which are enriched for the process “Arginine biosynthesis (p-value is 8.1E-28)”. The second module contains 8 operons (23 genes), and 22 genes are involved in “Sulfur metabolic processes (p-value is 2.1E-39)”. The results of other modules are shown in Table 4. Each module we identified has at least one transcription factor (except for module 4). We also found that the all of the overlapping nodes we discovered are of the first type. Besides gene *ecfI*, which doesn't have any annotation, the other overlapping nodes all share different functions across modules.

### Comparison with other methods

To test the performance of our algorithm, we compared our partitions with four popular algorithms: Infomap^35^, LPA^57^, Fastgreedy^51^ and Walktrap^55^. Empirical networks are compared using NMI values, as shown in table 2. The others are compared using the modularity Q metric, as shown in table 3. We can see that our algorithm, LPA and Walktrap all perform well on synthetic networks. However, for empirical real world networks, our algorithm and Infomap perform better than the other methods. Our algorithm and Fastgreedy always have stable higher modularity. We conclude that our algorithm performs well and is competitive with other methods.

## Discussion

Discovering complex network community structure has become an important challenge during the past decade. Several advanced algorithms have been proposed to detect community structures in complex networks, but each has limitations^35^. For example, some approaches don't perform well on large-scale networks, some need to pre-estimate community numbers, some can't uncover overlapping communities, some depend on multiple parameters, some unable to discover sparse modules or small communities, some are domain-specific, work with specific structures, and still some don't generate stable partitions etc.

Our algorithm overcomes most of these limitations. It's a parameter free approach, and can find communities from adjacency lists of nodes directly. Thus it is conceptually very simple, efficient, easily implementable and suitable for large-scale networks. As we have shown, it can be applied to networks from multiple domains. It can auto-detect the number of communities, discover isolated nodes and isolated communities, and always outputs stable partitions. Furthermore, It can offer two different kinds of overlapping communities, and detect cohesive communities, sparse communities and small communities as well. There are many sparse communities and small communities in real world networks, so it is important to be able to identify these structures. For example, sparse communities and small communities are important structures in biological networks.

We propose a novel and simple framework to detect community structure of complex networks based on network topology. Compared to popular methods previously reported in the literature, our algorithm preforms competitively for both synthetic and real networks, but as we all know, it is far from providing an unforeseen breakthrough in community finding. In the future, we intend to improve the ability of our algorithm to detect the second type of overlapping nodes and discover hierarchical structure of complex network. While in the current implementation we do identify most overlapping nodes, others are left out, hindering the ability to split bigger communities into smaller ones.

## Methods

We defined community based on three properties, and then propose a simple and novel framework to detect communities based on network topology. The three properties of community are community structure property, community membership property and overlapping member properties respectively. The community structure property is used to define a community, while the community membership and overlapping member properties are used to define the members of a community.

### Community structure property

Although there is not a general definition, it is widely accepted that a community should be a sub-network that is internally densely connected, while externally sparsely connected^23^,^24^,^26^,^27^,^31^. Here we define two types of community structures: strong sense and weak sense communities. A sub-network is defined as a strong sense community if its internal connections are larger than its external connections. A sub-network is defined as a weak sense community, if its internal connections are equal or smaller than its external connections, but its internal connections are larger than the connections between this sub-network and any other communities.

**Figure 3:**
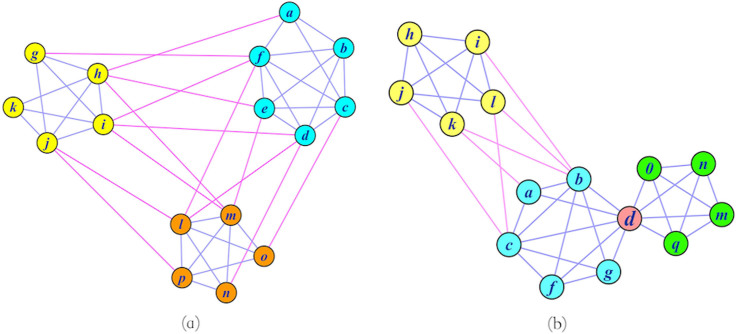
Example of community property. (a) Community structure property: the nodes of this network divide into three clusters, the cyan cluster is a strong sense community, and the others are weak sense communities. (b) Overlapping member property: node d is a second type of overlapping node shared by cyan community and green community.

In Fig. 3 (a) we provide an example of two types of community structures. In this network, the internal connections of a community are colored blue, and the connections among communities are colored purple. Based on our definition, it's easy to see that the cyan community is a strong sense community, as it has more internal connections than external connections. While the yellow community is a weak sense community, as although the number of internal connections equals the number of external ones, its internal connections are more numerous than the connections between it and any one of the other two communities. As we can see, the orange community is also a weak sense community.

### Community membership property

By definition, a member of a community should have more neighbors within its community than in any other community, unless it is an overlapping member. That is to say, each node should join the community, which has its maximum number of neighbors, except for overlapping nodes.

### Overlapping member property

We define two types of overlapping nodes. One is based on the number of connections between it and corresponding communities, and the shortest path between it and hub members of corresponding communities, while the other is based on topology structure of community. For the first type of overlapping node, not only the number of connections between it and the corresponding communities should be equal, but also the shortest path between it and hub members of corresponding communities should be equal as well. For the second type of overlapping node, it should be tightly connected with both communities, and there should be few connections between the two corresponding communities if remove such nodes. As the network in Fig. 3 (b) shows, node “d” should be assigned to the cyan community if we don't take into account overlapping member properties. However, node “d” is an overlapping node of the second type, connecting the cyan and green communities, based on our definition.

### Hub member

A hub member has the most neighbors within a community.

### Algorithm

**Figure 4:**
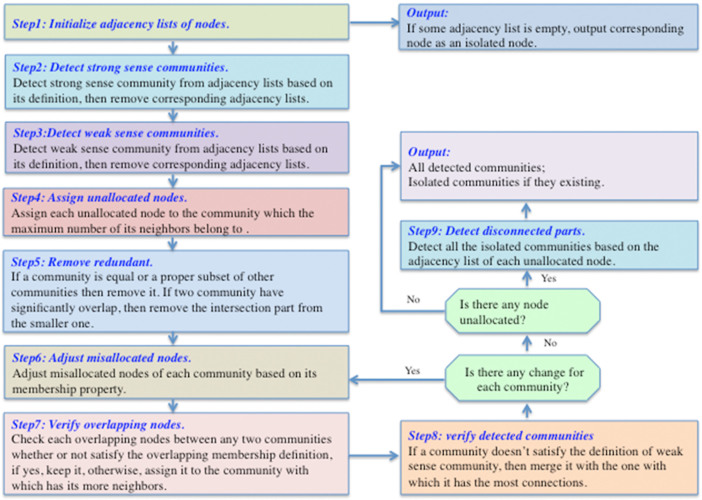
The flowchart of our algorithm. The flow chart described our algorithm step by step in detailed. It includes 8 main steps and 1 additional step if necessary.

Our algorithm is based on the idea that community structures can be detected from sub-networks by comparing the number of internal and external connections of each community, and it is mainly made up of four parts: initialize the adjacency lists of nodes, search for strong sense communities from adjacency lists, detect weak sense communities based on strong sense ones from adjacency lists, and iteratively readjust nodes to discovered communities based on their community membership property until the approach converges. The specific steps are as shown in Fig. 4. An example of the application of the algorithm to a network is shown in the supplementary materials.

Due to the fact that our algorithm is similar in spirit to the label propagation algorithm (LPA), we compare its performance with LPA. The greatest similarity between these two algorithms is that they both tend to assign each node in the network to the community with which they have the maximum number of neighbors. The main differences have to do with the fact that LPA initializes node communities, while we initialize edge communities (the adjacency list of each node denotes all it's corresponding edges); LPA depends on specific random seeds, initial conditions and tie-break rules for its execution, while our approach doesn't; Our algorithm is a deterministic algorithm, while LPA isn't; LPA searches for communities based on label dynamic propagation and static network topology, while our algorithm searches for community only based on static network topology.

### Evaluation measures

To evaluate the performance of our algorithm we need an approach to measure the accuracy of community partitions. However, there is no general standard technique for this, because it is difficult to know the structures of real world networks a priori, Therefore, we use the normalized mutual information (NMI) measure^16^ to evaluate community partitions. NMI is defined as follows 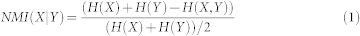
Where X corresponds to the real communities, Y corresponds to the predicted communities, and H(X) denotes the entropy of random community X, whereas H(X,Y) H(X,Y) denotes the joint entropy of X and Y.

For other networks we use the modularity^28^ measure to evaluate the quality of a partition. It is based on the intuitive idea that random networks do not exhibit community structure. Let us define a matrix e where the elements *e_ij_* represents the fraction of total connections between two different communities, and the real fraction of links exclusively within a community is *e_ii_* Then the sum of any row of e, 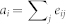 corresponds to the fraction of links connected to community *i*, and the expected number of intra-community links is just 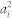. We can compare *e_ii_* and 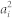 directly, and sum over all the communities in the network. This measure is known as modularity: 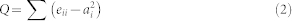
Complexity Analysis

It is advantageous for an algorithm to have lower time complexity, so that it can be applied to large-scale networks. Our algorithm consists of two stages: the first stage is detecting communities, and the cost time complexity is *O(k*^*2*^*+lkn),* where n is the number of nodes, *k* is the number of detected communities and *l* is the maximum size of the initial adjacency lists. The second stage is adjusting membership among communities, and has a cost time complexity *O(vk*^*2*^*+lk*^*2*^*+kn),* where *v* is the maximum count of overlapping nodes. Thus, the total time complexity for both steps is *O((v+l)k*^*2*^*+lkn).* As a result, this algorithm can be efficiently applied to a network of tens of thousands of nodes and the execution time is around an hour on a typical CPU.

## Author Contributions

W.L. analyzed data, designed and performed research. M.P., W.L. and X.F.W. discussed the results and wrote the manuscript text. All authors reviewed the manuscript.

## Supplementary Material

Supplementary Information Supplementary Information: Detecting Communities Based on Network Topology

## Figures and Tables

**Table 1:** The lists of we analyzed networks

Networks	Nodes	Edges	Reference
GR_256	256	702	[29]
LFR_128	128	597	[42]
LFR_256	256	1294	[42]
LFR_512	512	3285	[42]
Karate	34	78	[30]
Dolphins	62	159	[32]
Football	115	613	[14][31]
Pol-books	105	441	[14]
Jazz	198	2742	[36]
E. Coli	418	519	[33]
Email	1133	5451	[44]
Facebook	2888	2981	[34]
Protein	3274	8748	[43]
Power	4941	6594	[7]
Collaboration	5242	14490	[41]
PGP	10680	24316	[37]

**Table 2:** The analyze of empirical networks

Networks		Ours		Infomap		LPA		Fastgreedy		Walktrap	
Names	Clusters	N_c_	NMI	N_c_	NMI	N_c_	NMI	N_c_	NMI	N_c_	NMI
GR_256	18	18	1.0000	18	1.0000	18	1.0000	18	0.9688	18	0.9890
LFR_128	8	8	0.9184	50	0.6233	8	0.9187	7	0.8276	8	0.9200
LFR_256	14	14	0.9751	109	0.6776	14	0.9676	12	0.8498	14	0.9681
LFR_512	26	26	0.9820	219	0.7168	24	0.9304	17	0.7085	24	0.9172
Karate	2	2	1.0000	3	0.6995	3	0.6995	3	0.6925	5	0.5042
Dolphins	2	3	0.7621	6	0.5373	6	0.5699	4	0.5571	4	0.5816
Football	12	11	0.9492	12	0.9720	9	0.8947	6	0.7532	10	0.9369
Polbooks	3	3	0.5780	6	0.4935	3	0.5744	4	0.5308	4	0.5428

*N_c_* represents the number of communities detected by different algorithms, and clusters represent the number of real communities of empirical network.

**Table 3:** The analyze of unempirical networks

	Ours		Infomap		LPA		Fastgreedy		Walktrap	
Networks	N_c_	Q	N_c_	Q	N_c_	Q	N_c_	Q	N_c_	Q
Jazz	3	0.4397	7	0.2800	3	0.4295	4	0.4389	11	0.4384
E. Coli	46	0.8902	72	0.7351	55	0.7445	45	0.7784	52	0.7463
Email	19	0.5374	69	0.5260	5	0.0320	12	0.5070	49	0.5307
facebook	7	0.7307	11	0.7961	9	0.7496	8	0.8087	6	0.6331
Protein	303	0.8673	423	0.7105	365	0.7095	203	0.7632	395	0.7291
Power	632	0.8199	487	0.8182	495	0.8043	41	0.9335	364	0.8310
Collab.	684	0.8378	716	0.7921	720	0.7950	427	0.8026	815	0.7824
PGP	770	0.8194	1065	0.8014	985	0.8023	204	0.8525	1574	0.7894

*N_c_* represents the number of communities.

**Table 4:** The function annotations of E. coli. modules detected by our algorithm

I_m_	N_o_	N_g_	Function clusters	P-value	TFs	N_m_
1	7	10	Arginine biosynthesis	8.1E-28	1	10
2	8	23	Sulfur metabolic process	2.1E-39	2	22
3	6	10	Carbohydrate catabolic process	6.0E-12	2	9
4	4	6	Cellular carbohydrate metabolic process	5.6E-7	0	6
5	15	51	Locomotion	2.2E-90	2	45
6	12	16	SOS response	8.5E-29	1	13
7	14	27	Amine metabolic process	7.6E-22	2	22
8	7	7	Cellular amino acid biosynthetic process	3.6E-10	2	7
9	5	5	Cytoplasm	3.6E-3	1	5
10	5	23	Ion transport	5.0E-34	1	22
11	16	24	Nitrogen compound biosynthetic process	1.9E-19	1	19
12	16	18	Cellular process	3.1E-3	5	16
13	24	34	Cellular metabolic process	6.2E-6	1	25
14	18	35	Cellular metabolic process	5.8E-10	8	32
15	5	12	Chorismate metabolic process	3.0E-25	1	11
16	8	11	Cellular aromatic compound metabolic process	2.4E-15	1	10
17	24	56	Cellular process	7.3E-4	3	15
18	25	73	Generation of precursor metabolites and energy	1.7E-69	5	58
19	10	19	Macromolecule metabolic process	1.2E-5	1	15
20	124	236	Primary metabolic process	8.9E-21	35	183
21	6	7	Monocarboxylic acid metabolic process	1.1E-8	2	6
22	6	11	Carbohydrate metabolic process	1.1E-9	1	10
23	26	71	Metabolic process	1.1E-5	9	61

*I_m_* represents the index of Module, *N_o_* represents the number of operons each module contains, *N_g_* represents the number of genes each module contains, TFs represents the number of TF in each module, *N_m_* represents how many genes are matched with the DAVID *Escherichia coli* database in each module.
