# Supplementary material for: Detecting Communities Based on Network Topology
Source: Sci Rep. 2014 Jul 18;4:5739. doi: 10.1038/srep05739 (PMC4102921; doi:10.1038/srep05739)
Supplement: Supplementary Information — Detecting Communities Based on Network Topology [file srep05739-s1.pdf]

Supplementary Information: Detecting Communities Based on Network Topology

Wei Liu, Matteo Pellegrini, Xiao-Fan Wang

In order to explain our algorithm, we provide an example using the Karate Network, and a detailed description of each of these steps follows.

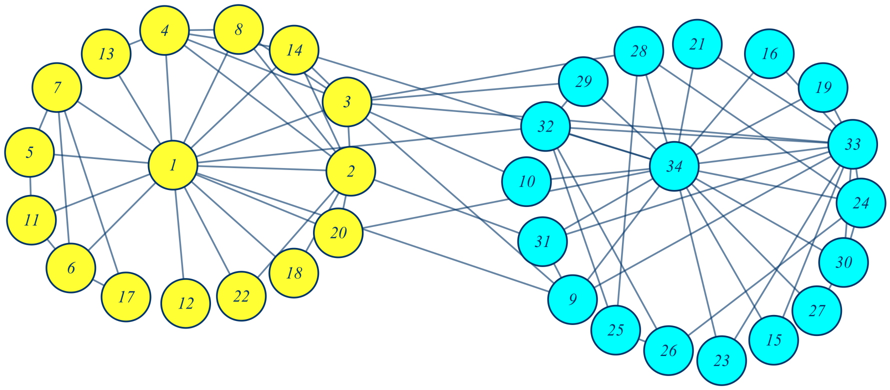

Figure.S1 The karate network

1. Algorithm: (Karate network)

Step1: Initialize adjacency lists of nodes.

For each node of the karate network, we initialize the corresponding adjacency list as shown below:

c{1}=[1, 2, 3, 4, 5, 6, 7, 8, 9, 11, 12, 13, 14, 18, 20, 22, 32];
c{2}=[1, 2, 3, 4, 8, 14, 18, 20, 22, 31];
c{3}=[1, 2, 3, 4, 8, 9, 10, 14, 28, 29, 33];
c{4}=[1, 2, 3, 4, 8, 13, 14];
c{5}=[1, 5, 7, 11];
c{6}=[1, 6, 7, 11, 17];
c{7}=[1, 5, 6, 7, 17];
c{8}=[1, 2, 3, 4, 8];
c{9}=[1, 3, 9, 31, 33, 34];
c{10}=[3, 10, 34];
c{11}=[1, 5, 6, 11];
c{12}=[1, 12];
c{13}=[1, 4, 13];
c{14}=[1, 2, 3, 4, 14, 34];
c{15}=[15, 33, 34];
c{16}=[16, 33, 34];

c{17}=[6, 7, 17];
c{18}=[1, 2, 18];
c{19}=[19, 33, 34];
c{20}=[1, 2, 20, 34];
c{21}=[21, 33, 34];
c{22}=[1, 2, 22];
c{23}=[23, 33, 34];
c{24}=[24, 26, 28, 30, 33, 34];
c{25}=[25, 26, 28, 32];
c{26}=[24, 25, 26, 32];
c{27}=[27, 30, 34];
c{28}=[3, 24, 25, 28, 34];
c{29}=[3, 29, 32, 34];
c{30}=[24, 27, 30, 33, 34]
c{31}=[2, 9, 31, 33, 34];
c{32}=[1, 25, 26, 29, 32, 33, 34];
c{33}=[3, 9, 15, 16, 19, 21, 23, 24, 30, 31, 32, 33, 34];
c{34}=[9, 10, 14, 15, 16, 19, 20, 21, 23, 24, 27, 28, 29, 30, 31, 32, 33, 34];

Step2: Detect Strong sense communities.

Compare the internal connections and external connections of every adjacency list c{*i*}, (*i*=1,…,34), If the former is larger than the latter, then define c{*i*} as a strong sense community. In this example, c{1}, c{2}, c{33} and c{34} are strong sense communities. We define these as candidate communities P{j} (j=1,…,4), and empty c{1}, c{2}, c{33} and c{34}.

Step3: Detect weak sense communities.

For each non-null c{*i*}, if its internal connections are larger than the maximum connections between it and every P{*j*}, then we take c{*i*} as a new candidate community and empty it. In this example, we didn't find any new candidate weak sense communities.

Step4: Assign unallocated nodes.

After the first three steps, if some nodes are still unallocated, then for each one we assign it to the candidate community, which has the most connections with it. In this example, nodes 17, 25 and 26 are initially unallocated. We assign node 17 to P{1}, and node 25 and 26 to P{4} based on their connections:

P{1}=[1, 2, 3, 4, 5, 6, 7, 8, 9, 11, 12, 13, 14, 17, 18, 20, 22,32];
P{2}=[1, 2, 3, 4, 8, 14, 18, 20, 22, 31];
P{3}=[3, 9, 15, 16, 19, 21, 23, 24, 30, 31, 32, 33, 34];
P{4}=[9, 10, 14, 15, 16, 19, 20, 21, 23, 24, 25, 26, 27, 28, 29, 30, 31, 32, 33, 34];

Step5: Delete redundant and significant overlapping communities.

For each candidate community, check whether it is equal, or a proper subset of other candidate communities. If it is, delete it. Otherwise, if two communities are significantly overlapping (if the overlapping nodes of two communities is larger than half the number of nodes of the smaller community), delete the intersection from the smaller one. In this example, no redundant communities exist, but P{1} is significantly overlapping with P{2}, as are P{3} and P{4}. After we delete the intersections, there remain three candidate communities:

P{1}=[1, 2, 4, 5, 6, 7, 8, 11, 12, 13, 14, 17, 18, 20, 22];
P{2}=[31];
P{4}=[3, 9, 10, 14, 15, 16, 19, 20, 21, 23, 24, 25, 26, 27, 28, 29, 30, 31, 32, 33, 34];

Step6: Adjust misallocated nodes.

Except for overlapping nodes, for each member of candidate community P{*j*}, compute the connections between it and every candidate community, and assign it to the community which has the most connections with it. In this example, node 31 is misallocated and is reassigned to P{4}.
Now there are only two candidate communities left:
P{1}=[1, 2, 4, 5, 6, 7, 8, 11, 12, 13, 14, 17, 18, 20, 22];
P{4}=[3, 9, 10, 14, 15, 16, 19, 20, 21, 23, 24, 25, 26, 27, 28, 29, 30, 31, 32, 33, 34];

Step7: Adjust overlapping nodes.

For each overlapping node between any two communities, if it has equal neighbors with the two communities and has equal distance with their corresponding hub nodes, we define it as an overlapping node. Otherwise, we assign it to the community with which it has the most neighbors. In this case, nodes 14 and 20 are overlapping nodes of two communities, and they are all assigned to P{1}. Now the result is:

P{1}=[1, 2, 4, 5, 6, 7, 8, 11, 12, 13, 14, 17, 18, 20, 22];
P{4}=[3, 9, 10, 15, 16, 19, 21, 23, 24, 25, 26, 27, 28, 29, 30, 31, 32, 33, 34];

Step8: Check whether candidate communities satisfy our definition.

For each candidate community, if it doesn't satisfy the weak sense community definition, merge it with the one with which it has the most connections. In this example, the remaining two candidate communities satisfy the community definition.

Because some members changed between the two communities, we need repeat step7 and step8 until there is no change in any community.

There are no unallocated nodes at this point, so we output the two final communities:

P{1}=[1, 2, 3, 4, 5, 6, 7, 8, 11, 12, 13, 14, 17, 18, 20, 22];
P{4}=[9, 10, 15, 16, 19, 21, 23, 24, 25, 26, 27, 28, 29, 30, 31, 32, 33, 34];

As shown in Fig.S1, P{1} is the yellow community, and P{4} is the cyan community.

2. Some complement explanation of algorithm

(1) Equal community

In Step 5, if we find some communities are equal, then we remove redundant ones, and then take the remaining one as a potential community.

(2) Significant overlap

In step 5, we take those community pairs that have more than two overlapping nodes as significant overlap communities.

(3) Strong sense and weak sense community

Here we only consider those adjacency lists that have more than two nodes. When we detect strong sense or weak sense communities, we only search them from local structure of network (adjacency lists of nodes).

(4) Isolated communities

The detected isolated communities are a part of isolated parts of the disconnected network, and they are all strong sense communities based on community definition. All these isolated communities have very sparse structures, and most of them are small ones.

(5) Verify detected communities

In step 8, we only check each community whether or not satisfy the definition of weak sense community, that's because strong sense community must satisfy the definition of weak sense community.
